# Infection of Wildlife by *Mycobacterium bovis* in France Assessment Through a National Surveillance System, Sylvatub

**DOI:** 10.3389/fvets.2018.00262

**Published:** 2018-10-30

**Authors:** Édouard Réveillaud, Stéphanie Desvaux, Maria-Laura Boschiroli, Jean Hars, Éva Faure, Alexandre Fediaevsky, Lisa Cavalerie, Fabrice Chevalier, Pierre Jabert, Sylvie Poliak, Isabelle Tourette, Pascal Hendrikx, Céline Richomme

**Affiliations:** ^1^Anses, Unit of Coordination and Support to Surveillance, Maisons-Alfort, France; ^2^French Hunting and Wildlife Agency (ONCFS), Studies and Research Department, Auffargis, France; ^3^University Paris-Est–Anses, French Reference Laboratory for Tuberculosis, Maisons-Alfort, France; ^4^National Hunters Federation (FNC), Issy-les-Moulineaux, France; ^5^French General Directorate for Food (DGAL), Animal Health Unit, Paris, France; ^6^French Association of Directors and Managers of Public Veterinary Laboratories of Analyses (Adilva), Paris, France; ^7^French National Federation of Animal Health Defense Associations (GDS France), Paris, France; ^8^Anses, Nancy Laboratory for Rabies and Wildlife, Malzéville, France

**Keywords:** bovine tuberculosis, *Mycobacterium bovis*, surveillance, wildlife, badger, wild boar, France

## Abstract

*Mycobacterium bovis* infection was first described in free-ranging wildlife in France in 2001, with subsequent detection in hunter-harvested ungulates and badgers in areas where outbreaks of bovine tuberculosis (TB) were also detected in cattle. Increasing concerns regarding TB in wildlife led the French General Directorate for Food (DGAL) and the main institutions involved in animal health and wildlife management, to establish a national surveillance system for TB in free-ranging wildlife. This surveillance system is known as “Sylvatub.” The system coordinates the activities of various national and local partners. The main goal of Sylvatub is to detect and monitor *M. bovis* infection in wildlife through a combination of passive and active surveillance protocols adapted to the estimated risk level in each area of the country. Event-base surveillance relies on *M. bovis* identification (molecular detection) (*i*) in gross lesions detected in hunter-harvested ungulates, (*ii*) in ungulates that are found dead or dying, and (*iii*) in road-killed badgers. Additional targeted surveillance in badgers, wild boars and red deer is implemented on samples from trapped or hunted animals in at-risk areas. With the exception of one unexplained case in a wild boar, *M. bovis* infection in free-living wildlife has always been detected in the vicinity of cattle TB outbreaks with the same genotype of the infectious *M. bovis* strains. Since 2012, *M. bovis* was actively monitored in these infected areas and detected mainly in badgers and wild boars with apparent infection rates of 4.57–5.14% and 2.37–3.04%, respectively depending of the diagnostic test used (culture or PCR), the period and according to areas. Sporadic infection has also been detected in red deer and roe deer. This surveillance has demonstrated that *M. bovis* infection, in different areas of France, involves a multi-host system including cattle and wildlife. However, infection rates are lower than those observed in badgers in the United Kingdom or in wild boars in Spain.

## Introduction

Wildlife can serve as a reservoir for multiple pathogens and may serve as a sentinel of the disease risk to humans and domestic animals. As a result, disease surveillance in wildlife is strongly recommended to provide data on the epidemiological role of wild animals and for the development of adapted disease control measures (1).

Bovine tuberculosis (TB) is a contagious and zoonotic disease caused by *Mycobacterium bovis*, and occasionally by *M. caprae* or *M. tuberculosis* (hereafter referred to as MTBC). This pathogen primarily infects cattle but can be transmitted to a wide range of host mammals, especially numerous wild animals such as Eurasian badgers (*Meles meles*), wild boars (*Sus scrofa*), red deer (*Cervus elaphus*), and roe deer (*Capreolus capreolus*) (2). In the United Kingdom (UK) and in Ireland, the Eurasian badger is considered as TB reservoir, as is the wild boar in Spain. These species are involved in the transmission of *M. bovis* to cattle (3–6).

France is officially declared TB-free since 2001 in the bovine population, because < 0.1% of cattle herds being infected annually. However, outbreaks still occur and the number of infected herds has increased since 2004 in certain parts of the country, especially in the South-West: Dordogne, Charente and Pyrénées-Atlantiques (French administrative division called departments) and in the East of France (Côte-d'Or) (7).

In France, TB in wild animals was first detected in 2001 in the Brotonne forest (Normandy) in hunter-harvested red deer exhibiting gross lesions. In 2006, despite control measures (culling), apparent prevalence rates in this forest reached 24% in red deer and 42% in wild boars, the closed environment and high density of wild ungulates were considered major risk factors to explain such high prevalence rates (8). Elsewhere in France, sporadic cases of TB infection have been detected in red deer and/or wild boar in several areas: Côte-d'Or (Burgundy region), Corsica, Pyrénées-Atlantiques, Dordogne, and Ariège in 2002, 2003, 2005, and 2010, respectively. The first cases in wild ungulates were systematically detected by carcass examination in hunter-harvested animals. Since then, event-based surveillance programs (also called passive surveillance) and targeted (or active) surveillance programs for the disease including the badger have been implemented in these areas. TB infection in badgers was initially detected in 2009 in Côte-d'Or (5.7%, *n* = 918 in the 2009–2011 period), then in 2010 in Dordogne and Charente (4.8%, *n* = 417 in 2010–2011). In wild boars, prevalence rates observed in 2008 reached locally 16.5% in Côte-d'Or and 4.4% in 2010–2011 in Dordogne (9). All these cases were detected in the vicinity of cattle outbreaks (10–12).

Increasing concern regarding the status of TB infections in wildlife led the French General Directorate for Food (DGAL) and the main institutions involved in animal health and wildlife management to establish a national surveillance system for TB in free-ranging wildlife: the “Sylvatub” system. This system coordinated by the French platform for epidemiological surveillance in animal health (ESA-Platform), was launched in September 2011. The main aims of Sylvatub are to detect TB in wildlife, to estimate and monitor infection levels in infected areas, to characterize *M. bovis* strains isolated from wildlife and to harmonize surveillance at the national level.

This article summarizes the key data collected on TB infection in France between 2011 and 2017 in badgers, wild boars, red deer, and roe deer. We describe the organization of the Sylvatub system and the findings in terms of TB prevalence in wild boars and badgers, and necropsy data gathered during event-based and targeted surveillance in the four species.

## Materials and methods

### Stakeholders and organization of sylvatub

**Figure 1:**
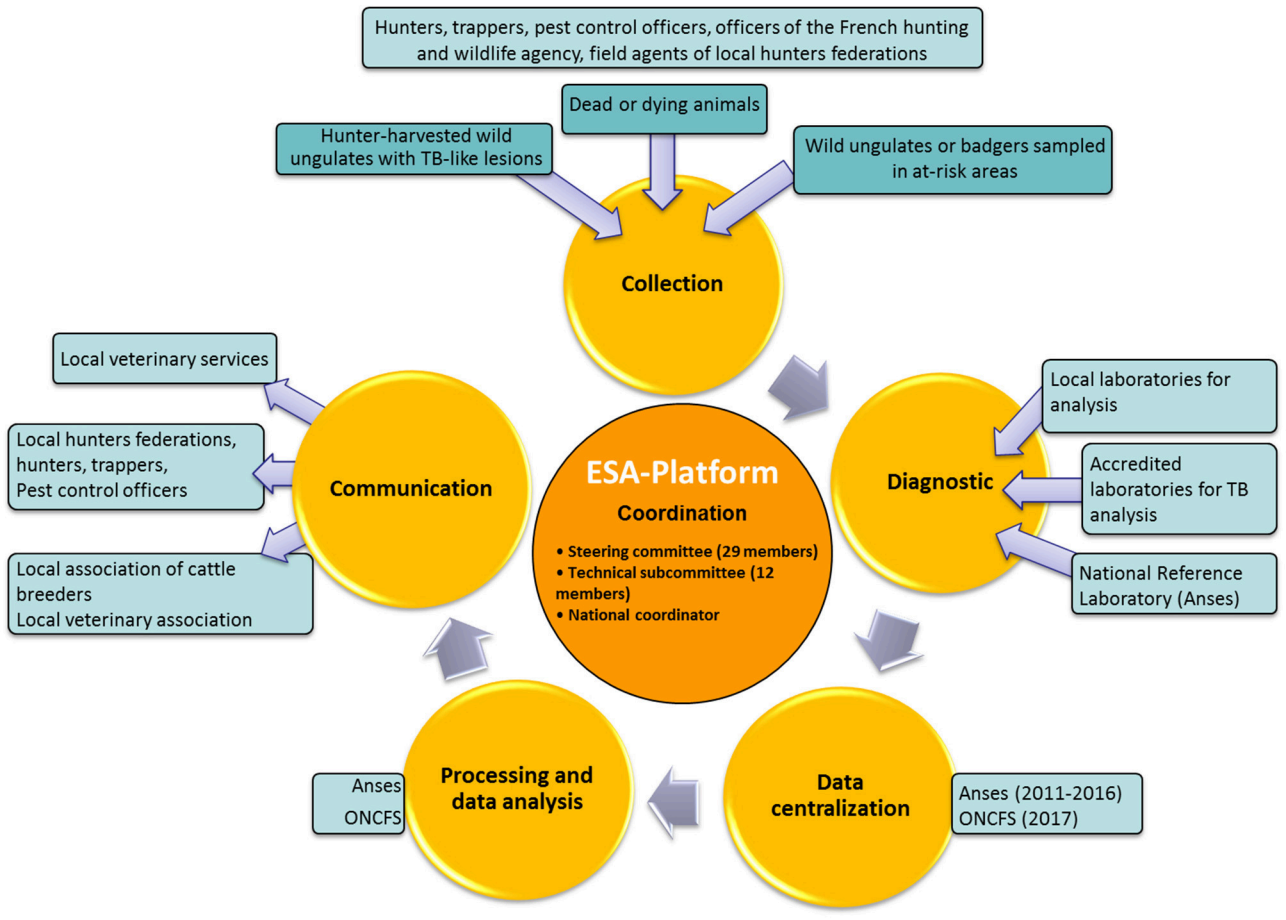
Simplified organization of Sylvatub. *Steering committee members*: French General Directorate for Food (DGAL), Ministry of the Environment (MEDDE), Regional Directorates for Food (DRAAF), Anses, French hunting and wildlife agency (ONCFS), National Hunters Federation (FNC), French association of pest control officers, French association of approved trappers, the French National Federation of Animal Health Defense Associations (GDS France), National veterinary association (SNGTV), Coop de France and French Association of Directors and Managers of Public Veterinary Laboratories of Analyzes (Adilva), Regional veterinary epidemiologists. *Technical subcommittee members*: DGAL, Anses, ONCFS, DRAAF, FNC, GDS France, Adilva.

The organizational structure of the system is shown in Figure 1 and was described by Rivière et al. (12). Briefly, the DGAL is in charge of the Sylvatub system. Coordination and technical operations are performed by the ESA-Platform www.plateforme-esa.fr. National governance is ensured by a steering committee and a technical subcommittee, where the different institutions or organizations involved in Sylvatub are represented (Figure 1).

The implementation of Sylvatub is based on the regular involvement of national stakeholders [national reference laboratory (NRL) for TB and the national coordinator of Sylvatub] and local stakeholders: veterinary services in charge of Sylvatub coordination, public administration in charge of the environment, hunting federations, associations of “*lieutenants de louveterie*” (historically called wolf-hunter, nowadays being state but volunteer officers in charge of pest control and who supervised badger trapping), trappers associations (volunteers who are duly trained and authorized), French hunting and wildlife agency services, local veterinary laboratories for animal health, cattle breeders, and finally veterinary associations.

### Sylvatub: a risk-based surveillance system

Sylvatub targets the wildlife species considered in 2011 to be the most relevant in the TB multi-host system: badgers, wild boars, red deer, and roe deer. Sylvatub's surveillance system components are based on event-based and targeted surveillance. These components are applied according to a risk-based surveillance approach, with three different levels of surveillance, applied at the level of French department (Table 1) and defined as follows:

- Level 3, for areas with several outbreaks in cattle and cases in wildlife. This level is applied for at least 4 years, to monitor infection levels in wildlife and to assess the efficacy of control measures;
- Level 2, for areas with sporadic at-risk outbreaks in cattle and/or areas in geographic proximity to high-risk areas. This level is applied for at least 1 year or for as long as necessary to develop a clear understanding of the epidemiological situation. A lower or higher surveillance level is subsequently applied, depending on the results obtained;
- Level 1, elsewhere in the country where no domestic and wild animal has been found infected for a long period of time.

**Table 1:** Surveillance methods implemented depending on the estimated risk level.

**Surveillance methods**	**Level 1**	**Level 2**	**Level 3**
**Event-based surveillance:**	X	X	X
- Detailed game carcass examination (wild ungulates)			
- SAGIR^(1)^ network (wild ungulates, badgers)			
**Strengthened event-based surveillance:**		X	X
- SAGIR network strengthened (red deer, wild boars, badgers)			
- Road-killed animals (badgers, wild ungulates)			
**Targeted surveillance in badgers in at-risk areas or around sporadic bovine outbreaks**		X	X
**Targeted surveillance in wild boars and red deer in at-risk areas**			X

(1) *Monitoring of dead or dying animals*.

The local surveillance level is re-evaluated twice a year, to align with the epidemiological situation in cattle and wildlife populations.

#### Event-based surveillance through detailed game carcass examination

This surveillance component is applied to all geographic areas, i.e., regardless of the local risk, to hunted wild boars, red deer, and roe deer. It is based on the analysis of animals with macroscopic TB-like lesions detected by hunters during post-mortem examination of all hunted games. The detection of gross lesions is supported by a national network of more than 55,000 hunters trained by the National Hunters Federation (FNC) for food safety purpose. Additionally to this framework, voluntary training courses were organized in the field to train hunters for recognition and reporting of TB-like lesions (internal abscesses or gross lymph nodes) and sampling of affected organs.

#### Event-based surveillance through the SAGIR network: wild animals found dead, moribund or with abnormal behavior

Event-based surveillance on dead and dying wild animals, through the SAGIR network (French hunting and wildlife agency/local hunters federations/FNC), has been implemented in France since 1986 (13). Within the Sylvatub system, dead animals belonging to TB-receptive species (badgers, wild boars, red, and roe deer) collected as part of the SAGIR network are tested for TB (*i*) in the presence of TB-like lesions for level 1 departments, and (*ii*) systematically for level 2 and 3 departments. Moreover, efforts are made to collect and test road-kill badgers in all the level 2 and 3 departments.

#### Targeted surveillance

Targeted surveillance may concern badgers, wild boars and/or red deer in level 3 departments depending on the population abundance and distribution, and badgers only in level 2 departments. This component of surveillance is implemented in “at-risk areas” determined as areas of about seven kilometers in radius around pastures of cattle outbreaks detected in the previous 4 years, and hunting or trapping locations of all infected wild animals. For badgers, at-risk areas are divided in two sub-areas: “infected area” (2 km radius) and “buffer area” (5 km radius around the infected area) to take into consideration badger home range which is smaller than in wild ungulates.

Sample sizes are determined to detect TB infection in at-risk areas, assuming a prevalence of 3%, with a 95% confidence interval. For wild boar and red deer, samples are defined for the whole at-risk area, whereas for badgers, one sample is for the infected area and one is for the buffer area. In large areas where wild populations could be considered as infinite, a sample of 130 animals per species is required. This sample size takes into account diagnostic test sensitivity (estimated at ~75% for PCR; see below). However, sample sizes are adjusted based on the surface of the area. In practice, samples between 60 and 260 red deer, wild boars, and badgers are programmed annually in each area. These are samples of hunted wild boars and deer or trapped badgers for control measures in infected areas.

Additionally, around sporadic cattle outbreaks outside at-risk areas (in level 2 and 3 departments), systematic TB analysis is conducted on a sample of about 15 badgers trapped within a radius of one or two kilometers depending on the number and localization of badger's setts. These small areas are called “prospecting areas.”

Animals are collected even if no macroscopic TB lesions are detected by field stakeholders.

### Tissue collection and laboratory investigations

#### Sample collection

Wild boars and red deer are collected by hunters, under the supervision of the local hunting federations during the hunting season (generally from August to March each year), and badgers are collected by trappers (accreditation required), under the supervision of pest control officers. In infected areas, where one of the control measures is to reduce badger populations, badger can be trapped, mostly from March to August. Field stakeholders (hunters, trappers, pest control officers) submit animals, organs, or tissues (from a standardized list of samples described in Tables 2, 3) directly to the local laboratory or store them in cooling rooms or freezers for later analysis. Data is collected for each animal on the species, estimated age (juvenile or adult), sex, date, location of collection and body condition (degradation, presence of lesions in the carcass, etc.). Age determination is based on animal size for badgers and/or weight and hunters' knowledge for wild ungulates. Trappers and pest control officers are volunteers but a financial compensation is provided for them.

**Table 2:** Diagnostic methods used in badgers from 2012 to 2017.

		**2012**	**2013**	**2014**	**2015**	**2016**	**2017**
	**Field samples**	**Entire carcass**					
Badgers	Pooled samples at local laboratory	- Retropharyngeal, tracheobronchial, mediastinal, hepatic lymph nodes and salivary glands; - Pool of lesions (if present)		- Retropharyngeal, tracheobronchial, mediastinal and hepatic lymph nodes - Pool of lesions (if present)			
	Analysis at local laboratory	Culture; PCR on pool of lesions			Culture; PCR on pool of lesions or PCR, culture on positive PCR pools	PCR; Culture on positive PCR pools	

**Table 3:** Diagnostic methods used in wild boars and deer for the hunting seasons from 2011 to 2017.

		**2011–2012**	**2012–2013**	**2013–2014**	**2014–2015**	**2015–2016**	**2016–2017**
Wild boars	Field samples	Head, pulmonary system, organs with lesions if present		Head, organs with lesions if present			
	Pooled samples at local laboratory	Cephalic and pulmonary lymph nodes; Pool of lesions (if present)		Submandibular lymph nodes; Pool of lesions (if present)			
	Analysis at local laboratory	Culture; PCR on pool of lesions				PCR; Culture on positive PCR pools	
Deer	Field samples	Head, pulmonary and digestive systems, organs with lesions if present					
	Pooled samples at laboratory	- Retropharyngeal, tracheobronchial, mediastinal and mesenteric lymph nodes: - Pool of lesions (if present)					
	Analysis at local laboratory	Culture; PCR on pool of lesions				PCR; Culture on positive PCR pools	

Tissue samples are taken in local laboratories even if no TB-like lesions are detected except for SAGIR network animals in level 1 department.

Tables 2, 3 shows the field samples collected for testing and changes over time. For wild boars, from mid-2013 until the present time, only the head has been collected and the submaxillary lymph nodes are tested. For badgers, which are relatively small, the entire animal is collected. Analyses at the local laboratory consist of post-mortem necropsy to detect TB-like lesions (caseo-granulomas, mineralized nodules, or purulent abscesses), polymerase chain reaction (PCR) and/or bacterial culture on pooled lymph node samples and on pooled samples with TB-like lesions.

As presented previously, diagnostic schemes differ between surveillance components: either TB testing is performed only if TB lesions are detected (SAGIR event-based surveillance in level 1 departments), or TB testing is performed systematically (for all suspect hunted carcasses and all SAGIR animals in level 2 and 3 departments, as well as for targeted surveillance in level 2 and 3 departments).

Analyses are performed as indicated in Tables 2, 3. The year 2015 marked a diagnostic-methodological transition for badger surveillance as these two diagnostic schemes were used depending on the local laboratory and the time of year. Changing from culture to PCR as a first line test was decided after having demonstrated that in cattle PCR provides a better sensitivity for TB detection without losing specificity (14).

#### Microbiological culture

Bacterial culture is performed following the protocol established by the French NRL (NF U 47–104) for isolation of *M. bovis*. Two to 5 g of sampled tissues were crushed with a 4% sulfuric acid solution to decontaminate the tissue. After 10 min, the acid was neutralized by adding a 6% sodium hydroxide solution. After decontamination, the supernatant was seeded on two different media: Löwenstein-Jensen and Coletsos. All seeded media were incubated at 37°C +/– 3°C for three months and exanimated every 2 weeks. If contamination is observed during the first month, samples are decontaminated a second time with 4% sodium hydroxide and then neutralized with 10% sulfuric acid solution.

The isolated *M. tuberculosis* complex (MTBC) colonies are confirmed by DNA amplification (15) targeting the IS*6110* sequence present in all species of MTBC (16), and *M. bovis* is confirmed by spoligotyping (see below).

#### Tissue PCRs

DNA extraction is performed on a pool of lymph nodes (retropharyngeal, pulmonary and mesenteric) and on organs with gross lesions when present, after mechanical lysis using an LSI MagVet™ Universal Isolation Kit (Life Technologies) with a KingFisher™ Flex automate (Thermo Scientific), following the manufacturer's instructions. The LSIV and MAX™ MTBC Real-Time PCR kit (Life Technologies), which targets IS*6110*, is used. A volume of 5 μL of the extracted DNA is mixed with 20 μL of reaction mix, and the reaction is carried out at 50°C for two min (1 cycle), followed by one cycle of 10 min at 95°C and 45 cycles of 15 s at 95°C, and one min at 60°C. Results are interpreted following the manufacturer's recommendations and by comParison with negative and positive controls. If DNA amplification is positive, *M. bovis* or any other MTBC species is confirmed by spoligotyping (see below).

#### Spoligotyping

Spoligotyping is performed as described by Zhang et al. (17), using TB-SPOL kits purchased from Beamedex® (Beamedex SAS, Orsay, France) on Bio-PLex 200/Luminex 200®. Molecular typing is performed either on MTBC isolates or directly on PCR-positive sample DNA. The presence or absence of the 43 spacer sequences contained in the DR locus is represented in a binary code of 43 entries. Spoligotypes are named according to an agreed international convention (www.mbovis.org).

### Data analysis

An infected animal is defined as an animal with an analytical result demonstrating *M. bovis* (or if it had been found *M. tuberculosis* or *M. caprae)* by molecular diagnosis or by bacterial culture.

All results presented in this article come from the Sylvatub national database.

Results are presented by calendar year for badgers and by 12 month period starting with the beginning of the wild boar hunt (August to the end of July). The Sylvatub system was set up in September 2011; as a result surveillance findings for wild ungulates are presented from the 2011–2012 hunting season to the 2016–2017 hunting season and from 2012 to 2017 for badgers.

**Figure 2:**
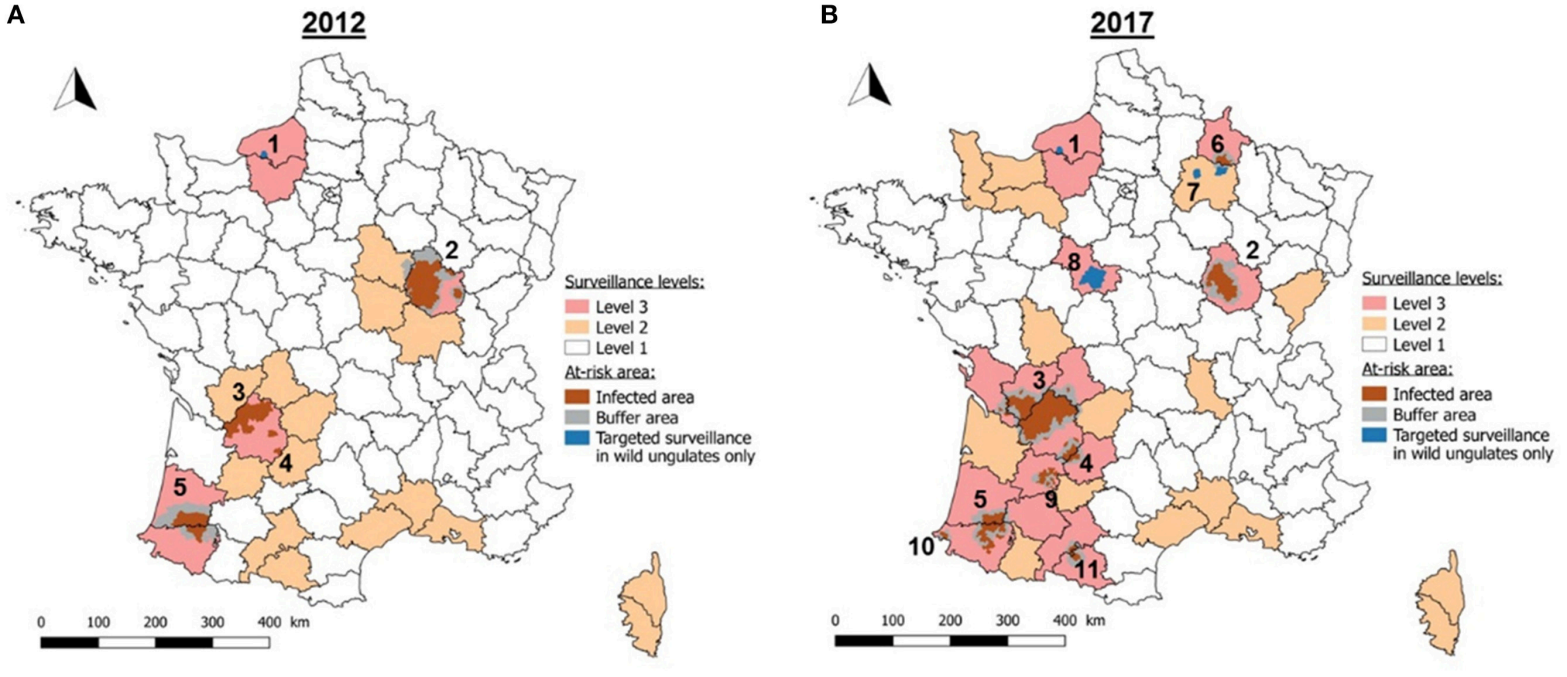
Changes in surveillance levels and at-risk areas between 2012 **(A)** and 2017 **(B)** (*1*: Brotonne-Mauny forest; *2*: Côte-d'Or; *3*: Dordogne/Charente/Charente-Maritime/Haute-Vienne/Corrèze/Gironde; *4*: Dordogne/Lot; *5*: Béarn; *6*: Ardennes/Marne; *7*: Marne (Reims Mountain); *8*: Loir-et-Cher (Sologne); *9*: Lot-et-Garonne; *10*: Pays Basque; *11*: Ariège/Haute-Garonne).

To simplify, at-risk areas are renamed with numbers as follows, by chronological order of detection of TB in wildlife: 1: Brotonne-Mauny forest (Seine-Maritime); 2: Côte-d'Or; 3: Dordogne/Charente/Charente-Maritime/Haute-Vienne/Corrèze/Gironde; 4: Dordogne/Lot; 5: Béarn (Pyrénées-Atlantiques/Landes/Gers); 6: Ardennes/Marne; 7: Marne (Reims mountain); 8: Loir-et-Cher (Sologne); 9: Lot-et-Garonne; 10: Pays Basque; and 11: Ariège/Haute-Garonne (Figure 2).

In this paper, apparent prevalence rates are indicated based on the diagnostic test used (culture or PCR). Furthermore, results were aggregated into two periods of 2 years each to calculate prevalence rates with more precision: P1 (2013 and 2014 for badger surveillance; the 2012-2013 and 2013-2014 hunting seasons for wild ungulate surveillance) and P2 (2016 and 2017 for badger surveillance; the 2015-2016 and 2016-2017 hunting seasons for wild ungulate surveillance). In P1, the diagnostic test used was sample culture, whereas in P2, PCR was used. We focused on areas where sampled animals were most numerous and where infection was confirmed to present and compare these results. To compare results in badgers between event-based and targeted surveillances, we focused in the at-risk area 3 because it is the only area where the number of analyzed badgers was sufficient for each of these surveillance components.

Regarding the number of analyzed animals, we counted only those with an interpretable analysis result (positive or negative). Apparent prevalence and 95% confidence intervals were calculated using exact binomial tests and *p*-values using the Fisher's exact test. Data analysis was performed using LibreOffice Calc (version 5.2) and R Studio software (version 3.3.1). Maps were generated using QGIS software (version 2.16.3).

## Results

### Functioning results

Since its implementation in 2012, Sylvatub has been gradually strengthened due to the larger number of areas where wild animals have been found infected, the enlargement of infected areas affecting cattle, and the detection of sporadic cattle outbreaks in new areas. This is reflected in the increase of number of departments at level 2 and 3 (from 21 in 2012 to 32 in 2017) (Figure 2). The surveillance levels for the departments were re-evaluated by the steering committee 10 times between 2011 and the end of 2017.

In 2012, there were five distinct at-risk areas. Since then, six other at-risk areas have been defined, with a total of 11 areas in 2017. Targeted surveillance was implemented for wild boars and red deer in three at-risk areas (numbered 1, 7, and 8), for wild boars and badgers in four at-risk areas (numbered 9, 5, 10, and 11) and for the three species in four at-risk areas (numbered 2, 3, 4, and 6) depending on the populations abundance and distribution.

In the meantime, size of these at-risk areas was expanded for five areas (3, 4, 6, 10, and 11), reduced for two areas (2 and 5) and remained approximately stable for the others (see details in Supplementary Table 1). The surface area of mainland France classified as at-risk was 14,397 km^2^ in 2012 and 20,434 km^2^ in 2017.

#### Event-based surveillance

The number of wild boars, red and roe deer collected by event-based surveillance increased from 77 in 2011–2012 to 134 in 2015–2016. In total, 316 wild boars, 197 roe deer, and 98 red deer have been collected since 2011. The number of dead or dying badgers collected per year (SAGIR and road-killed badgers) has increased from 70 badgers in 2012 to 582 in 2015. This number has been stable since 2015 with about 580 badgers collected per year, most of them being road-killed badgers.

#### Targeted surveillance

An average of 296 red deer (min: 226; max: 380), 1,420 wild boars (min: 1,078; max: 2,175) and 1,881 badgers (min: 1,447; max: 2,239) were analyzed per year between 2011 and 2017.

### TB infection in wildlife

#### TB infection in badgers

##### Event-based surveillance

**Figure 3:**
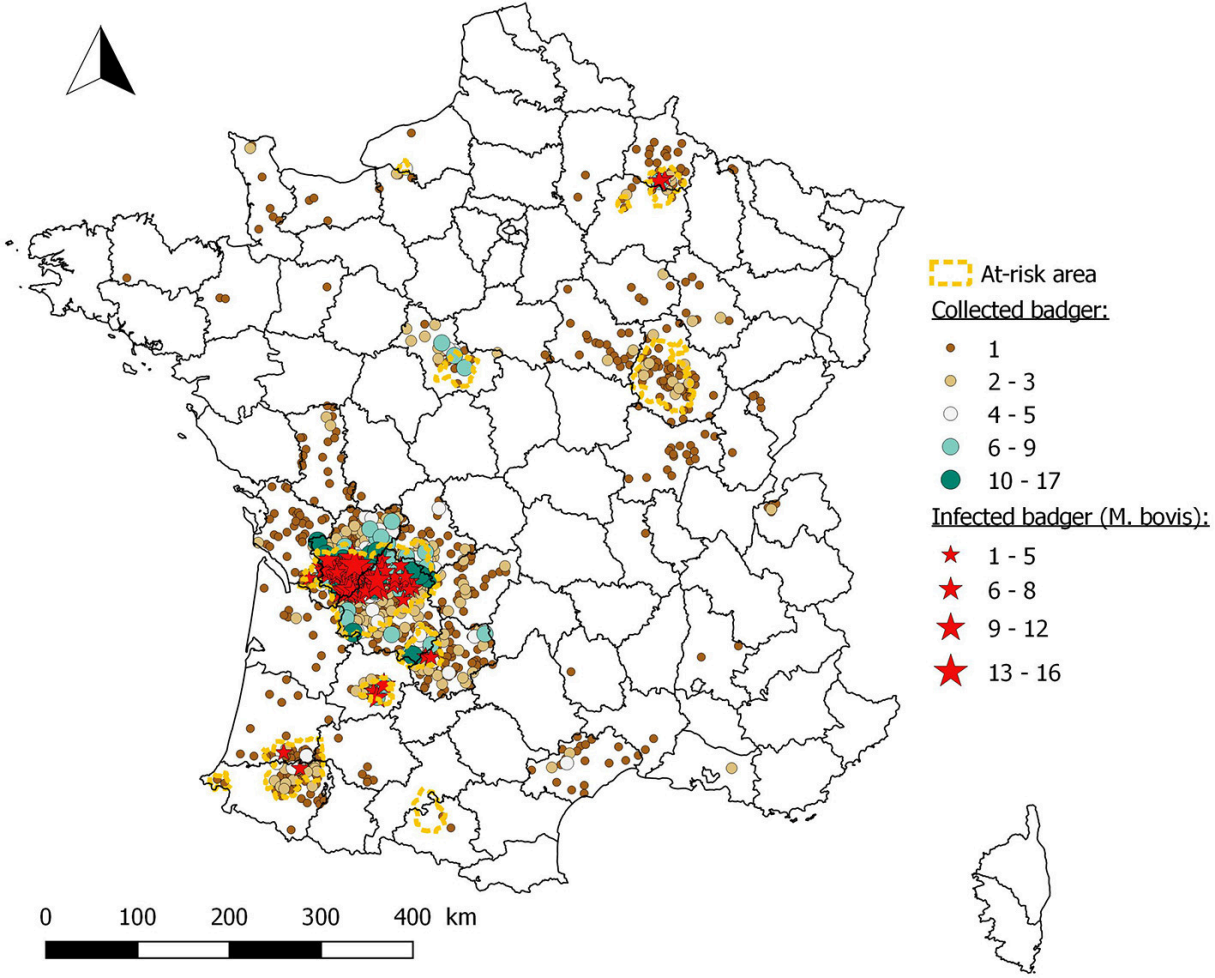
Location of badgers collected and found infected by event-based surveillance from 2012 to 2017.

Among the 2,491 badgers collected by event-based surveillance in 45 departments, 2,397 were analyzed (2,372 with an interpretable analysis result), and 89 were found infected with *M. bovis* (Figure 3). In all, 84 of these infected animals were found in the vicinity of cattle outbreaks (75 infected badgers in infected areas, five in buffer areas and four in prospecting areas). Five infected badgers (*n* = 716) were from outside but very close (<3.5 km) to at-risk areas (areas 3 and 9). In infected area 3, where badgers collected by event-based surveillance are numerous, apparent prevalence rates seems to be stable between P1 (8.2%; 95% CI: 4.2–14.2%) and P2 (9.6%; 95% CI: 6.8–13.1%).

##### Targeted surveillance

**Figure 4:**
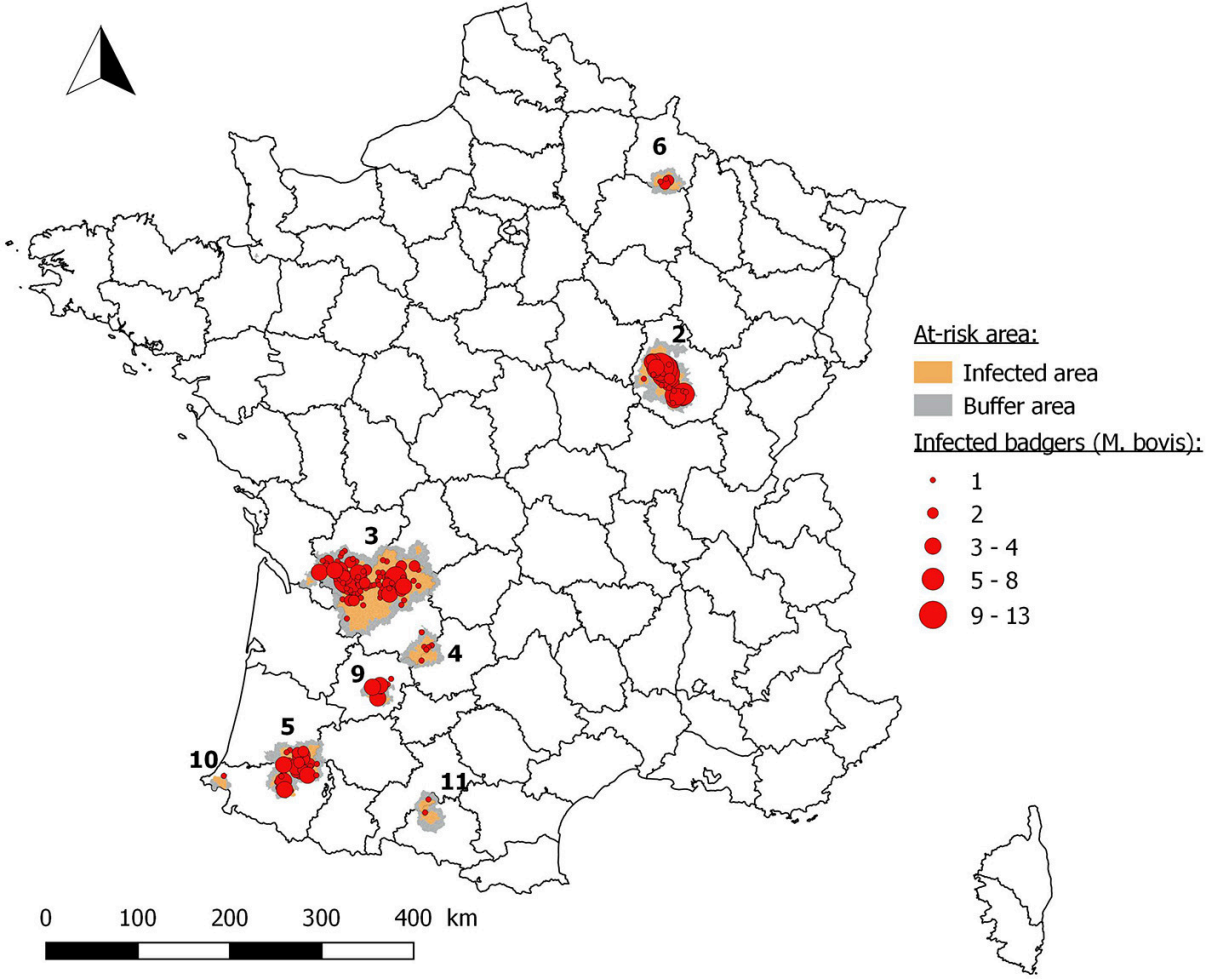
Location of infected badgers collected by targeted surveillance from 2012 to 2017 (*2*: Côte-d'Or; *3*: Dordogne/Charente/Charente-Maritime/Haute-Vienne/Corrèze/Gironde; *4*: Dordogne/Lot; *5*: Béarn; *6*: Ardennes/Marne; *9*: Lot-et-Garonne; *10*: Pays Basque; *11*: Ariège/Haute-Garonne).

From 2012 to 2017, 378 badgers (*n* = 10,184) were found infected with *M. bovis* by targeted surveillance in at-risk areas 2, 3, 4, 5, 6, 9, 10, and 11. In all, 340 of these badgers (*n* = 6,870) originated from infected areas and 27 (*n* = 3,314) from buffer areas (Figure 4). Targeted surveillance has not been implemented in at-risk areas 1, 7 and 8, where badger populations are very small.

In infected areas, apparent prevalence rate observed with culture (from 2012 to 2014) was on average 4.57% (*n* = 3,198) and with PCR (2016-2017) on average 5.14% (*n* = 2,412) (Supplementary Table 2). In buffer areas, apparent prevalence rate observed with culture was on average 1.33% (*n* = 1,508) and with PCR on average 0.41% (*n* = 1,217) (Supplementary Table 2). Regarding results in the four main infected areas (areas 2, 3, 5 and 6) for the two periods (P1 and P2), prevalence in badgers was significantly lower in P1 in infected area 3 than in the three other areas (*p* < 0.001; *p* = 0.009; *p* = 0.0351, respectively). In P2, prevalence was higher in infected area 5 than in areas 2 and 3 (*p* = 0.02; *p* = 0.013, respectively) (Table 4).

**Table 4:** Apparent prevalence rates in badgers collected by targeted surveillance in the four main infected areas of France between 2013-2014 (Period 1: P1) and 2016-2017 (Period 2: P2) [percentages are given with 95% confidence intervals (CI); in brackets number of infected/analyzed animals].

**No. of the infected area (full name of the area)**	**P1 (2013–2014) *Culture***	**P2 (2016–2017) *PCR***
	**Apparent prevalence rates [95% CI] (No. of infected badgers/no. analyzed)**	
2 (Côte-d'Or)	8.1% [6.3–10.3%] (61/751)	4.2% [2.6–6.2%] (22/528)
3 (Dordogne/Charente/Charente-Maritime/Haute-Vienne/Corrèze/Gironde)	2.7% [1.7–4.1%] (22/805)	5.3% [4.1–6.8%] (61/1143)
5 (Béarn)	5.9% [3.9–6.8%] (26/439)	7.9% [5.2–11.2%] (27/344)
6 (Ardennes/Marne)	6.7% [3.1–12.4%] (9/134)	3.1% [0.4–10.7%] (2/65)

In area 2, apparent prevalence was higher in P1 than in P2 (*p* = 0.008) (Table 4).

The targeted surveillance in prospecting areas has detected two sites of infection: in Ardennes in 2013, five infected badgers were detected from a total sample of 37 badgers collected close to four bovine TB outbreaks and, in 2015, in the Pays Basque (Pyrénées-Atlantiques), two infected badgers were detected from a sample of nine badgers, also sampled on the outskirts of four bovine TB outbreaks. As a result of these findings, infected and buffer areas were defined in these two departments and other cattle outbreaks were discovered nearby.

Males were found to have significantly higher infection rates (4.95%, *n* = 3,394) than females (2.02%, *n* = 4,213) (*p* < 0.001).

In infected area 3, prevalence were significantly higher (in P1 and P2) in badgers collected by event-based surveillance (mainly road-killed badgers) than by targeted surveillance (mainly trapped badgers) (*p* = 0.006 and *p* = 0.01, respectively) (Table 5).

**Table 5:** Comparison of apparent prevalence rates in badgers obtained by event-based surveillance and by targeted surveillance for two periods in infected area 3 [percentages are given with 95% confidence intervals (CI); in brackets number of infected/analyzed animals].

**Type of surveillance component**	**P1 (2013–2014) *Culture***	**P2 (2016–2017) *PCR***
	**Apparent prevalence rates [95% CI] (No. of infected badgers/no. analyzed)**	
Event-based surveillance	8.2% [4.2–14.2%] (11/134)	9.6% [6.8–13.1%] (35/365)
Targeted surveillance	2.7% [1.7–4.1%] (22/805)	5.3% [4.1–6.8%] (61/1143)

#### TB infection in wild boars and deer

##### Event-based surveillance

**Figure 5:**
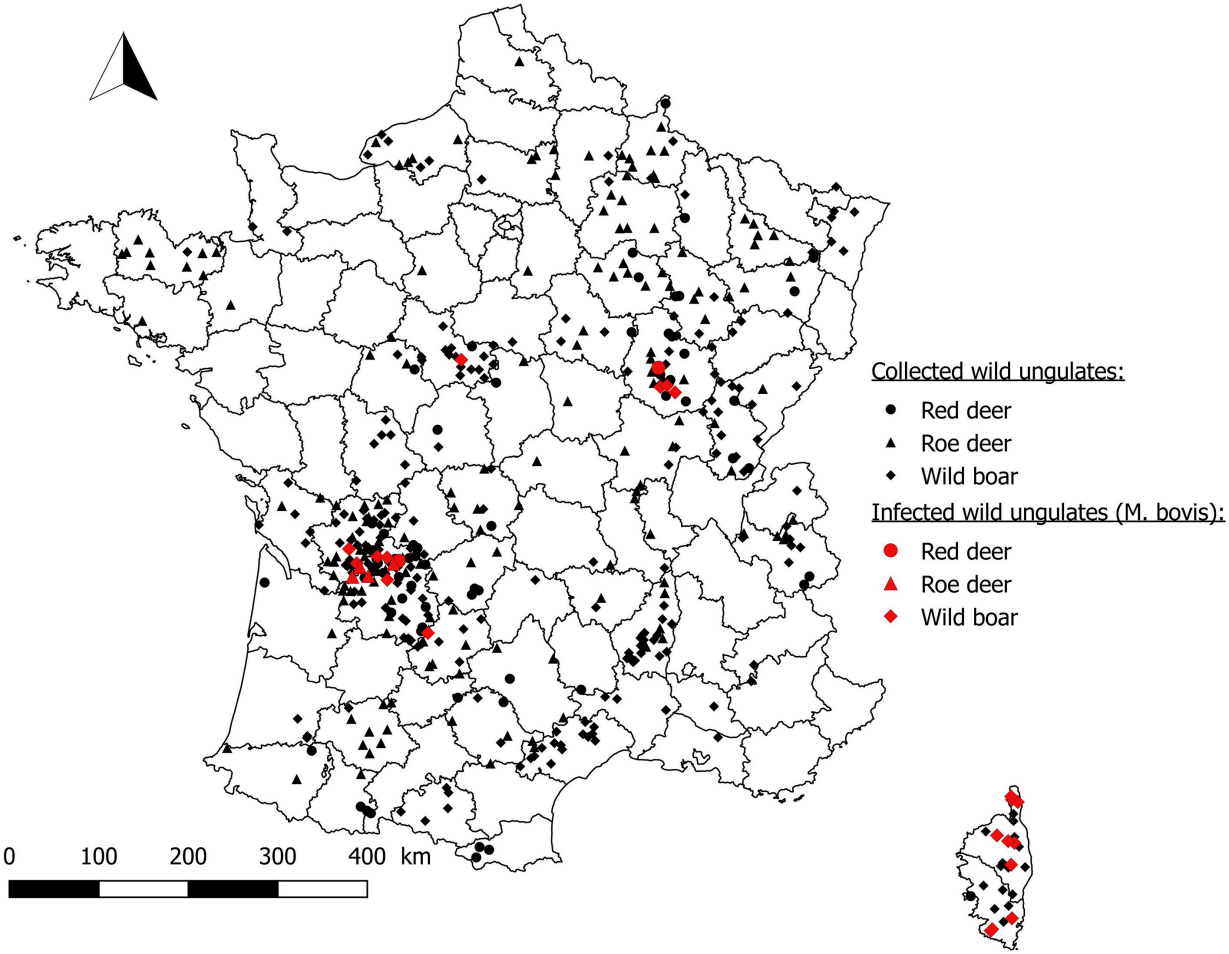
Location of wild ungulates collected by event-based surveillance from 2011 to 2017.

Among the 323 free ranging wild boars collected from 2011–2012 to 2016–2017 in 53 departments, 258 were analyzed, 241 had an interpretable analysis result and 29 were found to be infected in six departments (Dordogne, Charente, Côte-d'Or, Haute-Corse, Corse-du-Sud, and Loir-et-Cher). Ten came from at-risk areas (level 2 and 3 departments), 18 came from Corsica and one wild boar found infected in January 2015 in Loir-et-Cher, a livestock TB-free area since 1986 (Figure 5). This wild boar was found in open forest, although the area is surrounded by private game parks. *M. bovis* was also isolated from a wild boar in a closed game park in the Reims Mountain (Marne) in 2012 (18). Data for this case were not integrated in Sylvatub database because it was not a free ranging wildlife animal.

A total of 107 red deer and 190 roe deer have been collected through event-based surveillance since 2011 from 35 and 48 departments, respectively. Two red deer submitted by hunters both in 2016 were found to be infected in areas 2 and 3, and five infected roe deer were detected in area 3 in 2012, 2013, 2015, and 2016. For more details on roe deer surveillance in France see Lambert et al. (19) (Figure 5).

##### Targeted surveillance

Targeted surveillance on wild boars was implemented in 11 at-risk areas with 7,634 wild boars analyzed since 2011. In total, 180 wild boars were found infected with *M. bovis* in seven at-risk areas. In at-risk areas where infection in wild boars was known, apparent prevalence rate observed with culture (from 2011 to 2015) was on average 3.04% (*n* = 3,786), and with PCR (2015–2017) on average 2.37% (*n* = 2,536) (Supplementary Table 3). Prevalence in the main at-risk areas was between 1.5 and 4.3% in P1, and between 0.5 and 4.4% in P2 (Table 6). No infected wild boar has been found in areas 6, 7, 8, and 10 (Supplementary Table 3). In the Brotonne-Mauny forest (area 1) surveillance in wild boars has been implemented since 2001 due to the detection of infection at high prevalence rates in deer and wild boars (20). From that time point onwards, one to five additional infected wild boars were found each year between 2011 and 2017 (among about 200 wild boars analyzed/year) (Supplementary Table 3). Furthermore, following the discovery of one infected wild boar in Loir-et-Cher (Sologne), 986 wild boars were analyzed from 2015 to 2017 in open forest and in 12 game parks, but no additional wild boar was found to be infected.

**Table 6:** Apparent prevalence rates in wild boars in at-risk areas of France where infection has been found in wild boars in the 2012-2013-2014 period and the 2015-2016-2017 period [percentages are given with 95% confidence intervals (CI); in brackets number of infected/analyzed animals].

**No. of the at-risk area (full name of the area)**	**P1 (2012–2013 and 2013–2014) *Culture***	**P2 (2015–2016 and 2016–2017) *PCR***
	**Apparent prevalence rates [95% CI] (No. of infected wild boar/no. analyzed)**	
1 (Brotonne-Mauny forest)	1.5% [0.6–3.2%] (6/401)	1.3% [0.4–2.9%] (5/394)
2 (Côte-d'Or)	3.1% [1.7–5.1%] (15/483)	2.2% [1.0–4.2%] (9/404)
3 (Dordogne/Charente/Charente-Maritime/Haute-Vienne/Corrèze/Gironde)	4.1% [2.4–6.4%] (17/419)	2.7% [1.7–4.1%] (21/770)
4 (Dordogne/Lot)	4.3% [1.9–8.2%] (8/188)	3.2% [1.6–5.7%] (11/341)
5 (Béarn)	2.1% [0.9–4.2%] (8/373)	4.4% [2.4–7.4%] (13/295)
9 (Lot-et-Garonne)	/	4.2% [1.6–8.9%] (6/143)
11 (Ariège/Haute-Garonne)	/	0.5% [0–2.9%] (1/189)

*/: Targeted surveillance on badgers not required in the area*.

Infection in males and in females was similar (2.4%, *n* = 2,947 and 2.5%, *n* = 2,753, respectively) (*p* = 0.80).

Targeted surveillance on red deer was implemented in seven at-risk areas, where 1,817 red deer carcasses have been examined including 1,491 analyzed since 2011. Six red deer were found infected in area 2. Results by year and by area are detailed in Supplementary Table 4.

### *M. bovis* strains isolated in wildlife

**Figure 6:**
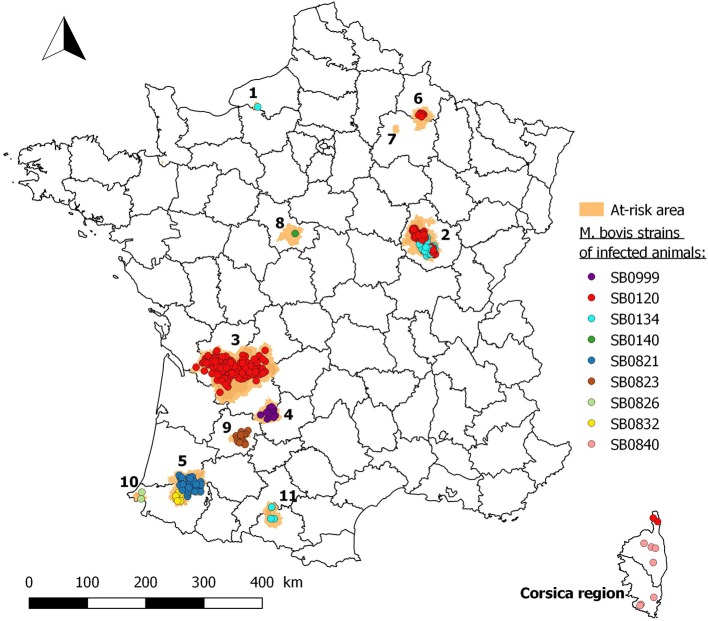
Location of *M. bovis* strains in wildlife in France (*1*: Brotonne-Mauny forest; *2*: Côte-d'Or; *3*: Dordogne/Charente/Charente-Maritime/Haute-Vienne/Corrèze/Gironde; *4*: Dordogne/Lot; *5*: Béarn; *6*: Ardennes/Marne; *7*: Marne (Reims Mountain); *8*: Loir-et-Cher (Sologne); *9*: Lot-et-Garonne; *10*: Pays Basque; *11*: Ariège/Haute-Garonne).

In total, nine genotypes of *M. bovis* have been identified in wildlife in France (Figure 6). Some of these genotypes are found in different at-risk areas (SB0120 in areas 2, 3, and 6 and in the Corsica region, SB134 in areas 1, 2, and 11), but their variable number tandem repeat (VNTR) profiles are different (Table 7). We should note the presence of two different genotypes in two areas: SB0120 and SB0134 in area 2, and SB0120 and SB0840 in Corsica. In area 5, the two genotypes SB0821 and SB0832 are in geographic proximity, but nevertheless in different sectors.

**Table 7:** Genotype of *M. bovis* strains in wildlife in France.

**No. of the at-risk area or region (full name of the area)**	**Spligotype**	**VNTR profile**
1 (Brotonne-Mauny forest)	SB0134	7 4 5 3 10 4 5 10
2	SB0120	5 5 4 1 11 4 5 6
(Côte-d'Or)		5 5 4 3 11 4 5 6
	SB0134	6 4 5 3 6 4 3 6
		6 5 5 3 6 4 3 6
3 (Dordogne/Charente/Charente-Maritime/Haute-Vienne/Corrèze/Gironde)	SB0120	5 3 5 3 9 4 5 6
4 (Dordogne/Lot)	SB0999	6 4 5 2 8 2 4 7
5	SB0821	6 5 5 3 11 2 5s 8
(Béarn)	SB0832	6 5 5 3 11 2 4s 8
6 (Ardennes/Marne)	SB0120	5 3 5 6 11 4 6 8
8 (Loir-et-Cher)	SB0140	7 5 6 3 10 3 4 7
9 (Lot-et-Garonne)	SB0823	6 5 5 3 11 2 5s 6
10 (Pays Basque)	SB0826	6 6 3 3 10 2 5s 8
11 (Ariège/Haute-Garonne)	SB0134	6 5 5 3 6 4 5 6
Corsica region	SB0840	7 4 5 3 8 2 5s 4
	SB0120	4 5 5 3 11 4 5 7

### Gross lesions and TB infection

#### Lesions in badgers

Among 357 badgers out of 13,620 (2.6%) necropsied showed TB-like lesions. Of these, 95 were found to be infected. Furthermore, only 21.5% of the 442 infected badgers showed TB-like lesions. Lesions were mostly found in the cephalic lymph nodes (retropharyngeal and submandibular) and the pulmonary tractus (lung, bronchial and mediastinal lymph nodes). In total, eight infected badgers had TB-like lesions on at least two internal organs and at least two lymph nodes. 16.2% of infected badgers (47/291) collected by targeted surveillance showed TB-like lesions, whereas in infected badgers collected by event-based surveillance, 31.0% (22/71) showed TB-like lesions (*p* = 0.04).

#### Lesions in wild boars

In all, 526 wild boars out of 7,838 (6.7%) collected by targeted surveillance showed TB-like lesions, and 77.8% of the infected wild boars showed TB-like lesions. Most of the TB-like lesions were found in the submandibular lymph nodes.

#### Lesions in deer

Among the eight red deer found infected since 2011, six came from targeted surveillance and two from event-based surveillance (submitted by hunters because of visible TB-like lesions). Five red deer showed gross TB-like lesions: in the pulmonary system for three red deer, in retropharyngeal lymph nodes for three, in mesenteric lymph nodes for one and in the liver for one. Gross TB lesions were also observed in infected roe deer (19).

## Discussion

### Organization of the surveillance system

Surveillance of TB in wildlife on a national scale in France, coordinated by the Sylvatub system, has been implemented gradually since 2011 thanks to the strong contribution of stakeholders at the national or local levels. Thanks to the involvement of hunters, event-based surveillance through detailed game carcass examination has often enabled us to detect first cases of TB in wild ungulates before targeted surveillance is deployed. It has help to detect TB as early as possible.

However, the main challenge for Sylvatub lies in the involvement of volunteer actors (hunters, trappers, pest control officers) and local veterinary services in such a complex multi-partner network. The targeted surveillance recommended in the Level 2 and 3 departments required a particularly strong involvement of local volunteers to collect tissue samples of wild ungulates and to trap badgers, explaining why some targeted plans have only been partially implemented in some areas. And these volunteers are, for the large majority, not those who are directly impacted by TB in cattle (cattle breeders) but could be affected by negative consequences if infection is found in wildlife, as for example, the implementation of density control measures of wild ungulates. Furthermore, in some departments, surveillance has been renewed for many years, which leads to a weariness of local actors. It should also be noted that the cost of Sylvatub is important for the government since it amounts to about 1 million euros per year.

### Sampling and diagnostic protocols

For each surveillance component, sampling was opportunistic and not performed randomly: for targeted surveillance it was based on trapper and hunter activity, and for event-based surveillance on different field actor interventions. Some areas or sectors were overrepresented whereas others were underrepresented depending on the number of volunteers, the intensity of their activity and the possibility to hunt. Moreover, samples collected were not strictly homogeneous from 1 year to the next, even within the same area.

For event-based surveillance, the collection of carcasses or the reporting of animals with TB-like lesions strongly depend on the awareness of actors in the field. We can reasonably expect that this awareness is generally greater for in the departments affected by TB (level 2 and 3 departments) which constitutes a selection bias.

In addition, wildlife surveillance must deal with unknown parameters such as densities, distribution, or the social behavior of animals. Surveillance must therefore adapt as well as possible to this variability and propose reasonable, realistic and attainable objectives but prevalence estimates are certainly biased. For example, it is difficult to set badger surveillance objectives without full knowledge of their densities and distribution. In practice, the samples planned in at-risk areas therefore had to be adjusted to the size of the area. We have set the design prevalence for sample size calculations at 3%. This threshold was chosen to be able to detect a relatively low prevalence while minimizing the sample size and therefore the cost. It could have affected the ability to detect TB in areas where TB is only present at prevalence below 3%, particularly in deer where prevalence seems lower than in the others species.

The amount of time dedicated to post-mortem examinations and their thoroughness influence the detection of visible lesions (5, 21). Here, wild ungulates were not always examined in a standardized way by hunters in the field. Even though training is provided, detection of lesions suggestive of TB by hunters in the field is less sensitive than the necropsy performed in the laboratory. Nevertheless, in some laboratories, necropsy of badgers or ungulates is sometimes performed without thoroughly inspecting all lymphatic nodes and organs. In addition, for wild ungulates, only organ blocks (head, lungs and mesenteric apparatus depending on the species and year) are transmitted to the local laboratories as part of targeted surveillance. For all these reasons, the frequency of lesions has certainly been underestimated.

Since 2011, the protocol for sampling biological tissue and the composition of analyzed pools of tissues have changed. Before 2014, for badgers, salivary glands were sampled and mixed with lymphatic nodes. For wild boars, since 2014, only the cephalic lymph nodes have been sampled and analyzed whereas before the pulmonary lymph nodes were also analyzed. The purpose of these changes was to concentrate the tissue pools with the organs most likely to be contaminated by *M. bovis*, in order to increase diagnostic sensitivity. Unfortunately, the impact of these measures could not be assessed (22, 23).

Moreover, the type of analysis used for TB diagnosis changed from 2015: bacterial culture was used from 2011 to 2014 and then, from 2015, PCR was deployed. The year 2015 was a year of transition with the use of culture or PCR, depending on the local laboratory. Sensitivities were estimated in cattle populations by Courcoul et al. (14): on average 87.7% [82.5–92.3%] for PCR vs. 78.1% [72.9–82.8%] for culture. Culture sensitivity is probably lower when used for wild animals due to field conditions and the potential contamination and degradation of samples and because only a limited range of tissues are collected and analyzed from each wild animal. Furthermore, pooled samples are analyzed for wildlife, whereas cattle samples are analyzed separately. All together these factors lead to a decreased culture sensitivity estimated by about 30–40% and PCR sensitivity by about 10–20% (Boschiroli, personal communication) compared to those in cattle.

In order to calculate prevalence more accurately, we grouped the results into two periods: P1 (2012–2014—use of culture) and P2 (2016–2017—use of PCR), and we calculated apparent prevalence. These prevalence rates do not take into account the sensitivity of the two analytical methods. For comparison of results in badgers, the year 2012 was not taken into consideration because it was the first year of operation of the Sylvatub system with a protocol that was not always fully implemented. The year 2015 was also ruled out because of the use of both analytical methods, depending on the local laboratory.

### *M. bovis* infection in wildlife

#### Distribution of infected wild animals

All infected wild animals detected since 2012 have been found in the vicinity of cattle outbreaks (in at-risk areas up to 10 km from recent cattle outbreaks pastures), except for one wild boar found in Loir-et-Cher (Sologne), an area without known TB infection. The molecular profile of the *M. bovis* strain (SB0140; VNTR profile: 7 5 6 3 10 3 4 7) found in this wild boar was identical to that of a bovine animal slaughtered in Vendée (western France) in 1997. This profile is currently unknown outside of France, which appears to indicate that the infection originated within the country. However, cattle monitoring revealed no cases in herds located or grazing within 5 km of where the infected wild boar was found (844 cattle tested using the comparative intradermal tuberculin test, all negative) (24). Moreover, targeted surveillance in wild boar and red deer populations from open and closed areas (game parks) in a perimeter of 12 km around the index case has been implemented since the 2015–2016 hunting season without any infected animal having being discovered. There is no clear evidence about the origin of this case to date but it seems essential that the greatest vigilance be given to game parks and associated game movements. Infected wild boars were found in all at-risk areas except in the area of Ardennes-Marne (area 6) which is the only area where no infected wild boars were found despite 4 years of targeted surveillance.

Infected badgers were mainly found in infected areas. A few were found in buffer areas (2–7 km from recent cattle outbreaks pastures) and only five from TB-free areas surrounding buffer areas. These five infected badgers were collected by event-based surveillance very close to at-risk areas (<3.5 km), and four of these five badgers were collected in 2012 and 2013 when perimeters of at-risk areas were not well defined. The discovery of these badgers has led to strengthened surveillance in cattle and subsequent detection of bovine outbreaks. However, the absence of TB detection in TB-free areas should be interpreted with caution due to the limited sampling size. In the UK, infected badgers were also detected between 1972 and 1993 in TB-free areas. In Ireland, this prevalence was estimated to be 15% in places where TB had not been reported in cattle for 6 years (25).

All isolates obtained from infected wild animals exhibited the same genotypes that had already been found in isolates from cattle outbreaks in the same regions. In areas where two different genotypes are isolated in wildlife, we observe exactly the same two genotypes in cattle. Strains with the SB0120 or the SB0134 spoligotypes are present in cattle and wildlife in different regions in France albeit presenting different VNTR profiles (26, 27). These results highlight the epidemiological relationship between wildlife and cattle, and evidence that *M. bovis* infection spreads within a multi-species system in these areas as also observed in the UK, Ireland and Spain (28–31). The current risk is that complex reservoirs of *M. bovis* including one or more wild populations and the environment are locally constituted. Badger and wild boar are at the moment the two species most found infected (see sanitary results) and thus worrying in terms of maintenance community. But recent finding on red foxes in France (32) and in other regions of continental Europe (33) raises questions about the epidemiological role of foxes, and have motivated ongoing investigations in different endemic areas.

#### Location of lesions

The location of lesions in badgers observed in France is consistent with previous studies carried out in the UK and in Ireland: the thoracic cavity (lungs and lymph nodes) and cephalic lymph nodes were the most common sites (3–5, 34, 35). As in these two countries, the presence of visible lesions only on lymph nodes was the most frequent finding.

With regard to wild boars, lesions are generally smaller and confined to the lymphatic nodes of the head (mainly submandibular lymph nodes) and are therefore less visible to hunters at the time of carcass examination. Systematic inspection of these lymph nodes in the laboratory or by a trained person is therefore essential.

The fact that the majority of infected deer have TB-like lesions and that these lesions are generally located in the pulmonary system makes it possible to suggest that surveillance by careful hunter examination and reporting observed lesions, remains a relevant and sensitive surveillance modality.

#### Prevalence of *M. bovis* infection

Concerning deer, we have observed only a few *M. bovis*-positive cases since 2012 (eight red deer and five roe deer) in France, all of them from Côte-d'Or and Dordogne/Charente (areas 2 and 3) which are infected areas with large deer populations, the presence of numerous cattle-TB outbreaks, confirmed infection in badgers and wild boars. These observations suggest a more minor role of deer in the interspecific transmission of *M. bovis* in France compared to other species such as wild boars or badgers. These results are similar to those obtained in others European countries, especially in the Czech Republic, in Hungary where sporadic cases were observed in red deer or in Poland and Italy in roe deer (36, 37). In Austria, red deer infected with *M. caprae* have been found since 1999 (38). In the UK, a large-scale study was conducted in 2007 and revealed a prevalence of 1.02% in red deer (*n* = 196) and 1.02% in roe deer (*n* = 885) (39).

The epidemiological situation of the Brotonne-Mauny forest (area 1) is special for France because of the very high prevalence level in red deer which were considered a TB maintenance host in the early 2000s (8, 9). This situation was also observed in southern Spain (*P* = 27.4%, *n* = 95 in the Doñana National Park) (40) and in Portugal between 2009 and 2013 (*P* = 38.3%, *n* = 115) (33).

The wild boar is considered a key maintenance host for tuberculosis in Spain with prevalence >50% in areas with high-density populations, such as in the Doñana National Park or in large hunting parks (22, 41). In Portugal, prevalence rates ranging from 6 to 46% have been observed (bacterial culture on a pool of lymph nodes) (42). Infected wild boars are commonly discovered in Germany, Italy and in several countries of Central Europe (43, 44).

In France, prevalence in wild boars is mostly lower [on average 3.04% in the 2011–2015 period (with culture) and 2.37% in the 2016–2017 period (with PCR)] than that observed in badgers in the same areas. This epidemiological situation is very different from that observed in south and central Spain where the wild boar is considered a key maintenance host for tuberculosis with prevalence >50% in areas with high-density populations, such as in the Doñana National Park or in large hunting parks (22, 41). In Portugal, prevalence rates ranging from 6 to 46% have been observed (bacterial culture on a pool of lymph nodes) (42). Infected wild boars are also commonly discovered in Germany, Italy and in several countries of Central Europe (43, 44).

Finally, outside the Sylvatub system, TB was also detected in 2012 in 7.3% of a wild boar population from a game park in the Marne, a cattle TB-free area (18). This detection raised questions on disease surveillance in captive wild animals, especially in game parks.

The prevalence observed in badgers in French infected areas was lower than that found in the bovine infection areas in the UK and Ireland. The prevalence rates estimated by culture in the UK during the randomized badgers culling trial, carried out between 1998 and 2006, varied widely (1.6–37.2%) depending on the *post-mortem* and culture methods used, with an average of 16.6% (3). In Ireland, 19.5 to 26.1% of the badgers analyzed by culture in the four study areas were found to be positive (45). A more recent study, in areas where there is high prevalence of bovine TB based on detailed *post-mortem* examination, histopathology, and culture in each specimen, reported a prevalence of 36.3% (4). Analysis of prevalence rates in ~5,000 badgers in Ireland revealed a decrease in the overall prevalence from 26 to 11% between 2007 and 2011 (46). In parallel, it should be noted that the herd prevalence in cattle in the UK and in Ireland is higher than in France (5–6 vs. 0.04%), and that all the analyzed badgers in these countries originated from areas with the highest prevalence in cattle (47).

In the north of Spain, in the provinces of Galicia and Asturias, where TB prevalence in cattle is between 0 and 4.3% depending on the district, badger prevalence rates between 6 and 7% are observed (28), which is similar to rates observed in some areas of France.

It is not possible to draw any conclusion on the relationship between the prevalence of TB in cattle herds and in surrounding badgers population due to important methodological differences. However, it would be also interesting to follow the relationship between the trends observed in some areas in cattle associated with disease control measures and the trend in the prevalence of these wild animal populations. Although the present dataset does not allow precise time comparison between P1 and P2 due to variations of sampling patterns and method for analyses, the ongoing surveillance may contribute to constitute consistent time series that may be analyzed for that concern. Nevertheless, in Côte-d'Or (area 2), if we considered that the apparent prevalence in P1 is underestimated because of the average sensitivity of the culture compared to that of the PCR, one can hypothesize that the prevalence has decreased in badgers from P1 to P2.

In Dordogne/Charente (area 3), where both event-based and targeted surveillance in badgers have been effective, we observe that badgers collected by event-based surveillance (mainly road-killed badgers) were more infected than those trapped. This could be explained by a lower vigilance of infected badgers and therefore increased collisions with vehicles. Moreover, movements of infected badgers are higher (48) and these infected badgers occupy larger home ranges (49, 50). These elements point to a greater probability of detecting infection by collecting road-killed individuals than by trapping.

Finally, we observed a higher prevalence in male than in female badgers, which corroborates several studies (51, 52). This has been interpreted as an influence of the aggressive behavior of males for the defense of the territories, and the attendance of several social groups during the rut period, resulting in a higher rate of infection in male badgers (52).

## Conclusion

The implementation of a wide and important surveillance system as Sylvatub relies on the involvement, at the national or departmental level, of the main organizations involved in wildlife surveillance and field volunteers without whom this surveillance would not be possible. The detection of numerous cases of TB in free-ranging wildlife occurs in areas of cattle outbreaks with the same profile of *M. bovis* strains showing evidence that, in main endemic regions of France, TB circulates between cattle and wildlife. The current risk is that complex reservoirs of *M. bovis* including one or more wild populations and the environment are locally constituted. It is also worrisome to note the increase in the number of areas in which infected badgers or wild boars are found, partly due to a better surveillance over time. However, prevalence observed in France in badgers and wild boars are lower than those observed in the UK and in Ireland for badgers or in Spain for wild boars, and only sporadic cases have been detected in red deer and roe deer.

Wildlife surveillance contributes to the implementation of control strategies in wildlife and in cattle by allowing defining at-risk areas. It also allows adapting surveillance in cattle keeping in mind the multi-host aspect of the disease as well as targeting prevention actions and to follow their long-term efficiency. This information, complemented by scientific investigations and researches, are needed for conducting biosecurity measures in wildlife (control of artificial feeding, management of hunting waste, banning game release, wildlife populations reduction in highly infected TB areas), and in livestock (management of water points, protection of the food and barns for example). Convinced that wildlife can be an additional local factor of TB spread and maintenance, the French ministry in charge of agriculture has decided to make Sylvatub sustainable.

In parallel, evolvement of sampling strategies have been discussed in the aim to improve surveillance. In buffer areas, targeted surveillance will be replaced by *M bovis* detection in road-killed badgers. Another main development will concern wild boar in at-risk areas: serological testing for the detection of antibodies directed against *M. bovis* have been proposed as suitable tools for TB screening in wild boar populations (18, 53). ELISA method has been tested in comParison to PCR and culture in different areas in France with encouraging results (Richomme and Boschiroli, personal communication). It will be used as an alternative method to monitor the *M. bovis* exposure level in wild boars in the next years.

## Ethics statement

Samples were collected from animals trapped with appropriate permits, hunted legally during the hunting season with appropriate permits, shot legally because of severe debilitation, or found dead. All the samples included in this study were obtained from animals analyzed within an official context relating to TB surveillance in free-ranging wildlife. All sampling procedures complied with national and European regulations, and no specific ethics approval was therefore required.

## Author contributions

JH, AF, ÉR, CR, M-LB, ÉF, LC, FC, IT, SP, and PH: Sylvatub conception and monitoring; ÉR: Data analysis; ÉR, M-LB, CR, and SD: Paper writing; CR, M-LB, SD, JH, ÉF, AF, LC, PJ, IT, SP, PH, and FC: Manuscript critical revision.

### Conflict of interest statement

The authors declare that the research was conducted in the absence of any commercial or financial relationships that could be construed as a potential conflict of interest.
