# Supplementary material for: Infection of Wildlife by Mycobacterium bovis in France Assessment Through a National Surveillance System, Sylvatub
Source: Front Vet Sci. 2018 Oct 30;5:262. doi: 10.3389/fvets.2018.00262 (PMC6220493; doi:10.3389/fvets.2018.00262)
Supplement: Supplementary file 1 [file Table_1.doc]

**SUPPLEMENTARY TABLE 1** Surfaces of each area from 2012 to 2017 (km²)

| **No. of the at-risk area**  **(full name of the area)** | **Type of area** | **2012** | **2013** | **2014** | **2015** | **2016** | **2017** |
| --- | --- | --- | --- | --- | --- | --- | --- |
| 1  (Brotonne-Mauny forest) | at-risk area | 82 | 82 | 82 | 82 | 82 | 82 |
| 2  (Côte-d’Or) | infected area | 3912 | 3035 | 2963 | 1940 | 1940 | 1978 |
|  | buffer area | 3169 | 1477 | 1434 | 1304 | 1343 | 1424 |
|  | total at-risk area | 7081 | 4512 | 4397 | 3244 | 3283 | 3402 |
| 3  (Dordogne/Charente/Charente-Maritime/Haute-Vienne/Corrèze/Gironde) | infected area | 2492 | 2188 | 3140 | 5657 | 6097 | 5842 |
|  | buffer area | / | 1592 | 2358 | 2835 | 3697 | 3549 |
|  | total at-risk area | 2492 | 3780 | 5498 | 8492 | 9794 | 9391 |
| 4  (Dordogne/Lot) | infected area | 95 | 80 | 69 | 303 | 493 | 570 |
|  | buffer area | / | 245 | 256 | 531 | 657 | 767 |
|  | total at-risk area | 95 | 325 | 325 | 834 | 1150 | 1337 |
| 5  (Béarn) | infected area | 2091 | 559 | 934 | 1347 | 1490 | 1606 |
|  | buffer area | 2556 | 1773 | 1533 | 1302 | 1553 | 1347 |
|  | total at-risk area l | 4647 | 2332 | 2467 | 2649 | 3043 | 2953 |
| 6  (Ardennes/Marne) | infected area | / | 242 | 269 | 300 | 364 | 364 |
|  | buffer area | / | / | 520 | 564 | 487 | 487 |
|  | total at-risk area |  | 242 | 789 | 864 | 851 | 851 |
| 7  (Marne - Reims mountain) | at-risk area | / | 200 | 200 | 200 | / | / |
| 8  (Loir-et-Cher) | at-risk area | / | / | / | 1042 | 1042 | / |
| 9  (Lot-et-Garonne) | infected area | / | / | 359 | 397 | 305 | 342 |
|  | buffer area | / | / | 333 | 490 | 513 | 343 |
|  | total at-risk area |  |  | 692 | 887 | 818 | 685 |
| 10  (Pays Basque) | infected area | / | 725 | 106 | 107 | 301 | 364 |
|  | buffer area | / | / | / | / | 333 | 691 |
|  | total at-risk area |  | 725 | 106 | 107 | 634 | 1055 |
| 11  (Ariège/Haute-Garonne) | infected area | / | / | / | 126 | 61 | 126 |
|  | buffer area | / | / | / | / | 303 | 554 |
|  | total at-risk area | / | / | / | 126 | 364 | 680 |

*/: Area not defined*
