# Supplementary material for: Infection of Wildlife by Mycobacterium bovis in France Assessment Through a National Surveillance System, Sylvatub
Source: Front Vet Sci. 2018 Oct 30;5:262. doi: 10.3389/fvets.2018.00262 (PMC6220493; doi:10.3389/fvets.2018.00262)
Supplement: Supplementary file 2 [file Table_2.doc]

**SUPPLEMENTARY TABLE 2** Apparent prevalence rates in badgers collected by targeted surveillance by area from 2012 to 2017 (percentages are given with 95% confidence intervals (CI); in brackets number of infected/analyzed animals)

| **No. of the at-risk area**  **(full name of the area)** | **Type of area** | **2012** | **2013** | **2014** | **2015** | **2016** | **2017** |
| --- | --- | --- | --- | --- | --- | --- | --- |
| 2  (Côte-d’Or) | Infected area | 3.0% [1.4-5.6%]  (9/300) | 11.3% [7.7-15.9%]  (29/256) | 6.5% [4.5-9.0%]  (32/495) | 6.1% [3.8-9.2%]  (21/343) | 5.3% [3.1-8.5%]  (16/302) | 2.7% [1.0-5.7%]  (6/226) |
|  | Buffer area | 0.2% [0-1.3%]  (1/424) | 0 [0-9.5%]  (0/37) | 0 [0-1.8%]  (0/203) | 0 [0-4.6%]  (0/78) | 0 [0-3.8%]  (0/96) | 0 [0-6.9%]  (0/52) |
| 3  (Dordogne/Charente/Charente-Maritime/Haute-Vienne/Corrèze/Gironde) | Infected area | 5.3% [2.9-8.7%]  (14/265) | 2.2% [0.9-4.2%]  (8/367) | 3.2% [1.8-5.3%]  (14/438) | 5.8% [4.1-8.0%]  (35/604) | 4.2% [2.7-6.2%]  (23/551) | 6.4% [4.6-8.7%]  (38/591) |
|  | Buffer area | 1.5% [0.2-5.2%]  (2/136) | 1.8% [0.2-6.2%]  (2/113) | 4.4% [2.1-8.3%]  (9/201) | 1.6% [0.5-3.8%]  (5/304) | 0.5%  [0 -2.7%]  (1/204) | 0.3% [0.1-2.5%]  (1/298) |
| 4  (Dordogne/Lot) | Infected area | 0  [0-17.6%]  (0/19) | 9.5% [1.2-30.4%]  (2/21) | 0  [0-24.7%]  (0/13) | 0  [0-17.6%]  (0/19) | 1.9% [0.2-6.7%]  (2/105) | 1.4%  [0-7.5%]  (1/72) |
|  | Buffer area | 0  [0-84.2%] (0/2) | 0  [0-18.5%] (0/18) | 0  [0-6.8%] (0/52) | 0  [0-3.2%] (0/113) | 0  [0-3.1%]  (0/116) | 1.0%  [0-5.6%]  (1/98) |
| 5  (Béarn) | Infected area | 0.4% [0-2.1%]  (1/267) | 5.7% [3.1-9.5%]  (13/229) | 6.2% [3.3-10.4%]  (13/210) | 3.6% [1.3-7.7%]  (6/165) | 9.0% [5.6-13.5%]  (20/223) | 5.8% [24-11.6%]  (7/121) |
|  | Buffer area | / | 2.2% [0.6-5.5%]  (4/184) | 1.9% [0.2-6.5%]  (2/108) | 0  [0-13.2%] (0/26) | 0  [0-3.0%]  (0/123) | 0  [0-3.7%]  (0/98) |
| 6  (Ardennes/Marne) | Infected area | / | 10.9% [3.6-23.6%]  (5/46) | 4.5% [1.3-11.2%]  (4/88) | 0  [0-7.7%]  (0/46) | 3.0% [0.1-15.8%] (1/33) | 2.9% [0.1-14.9%] (1/35) |
|  | Buffer area | / | / | 0  [0-14.2%]  (0/24) | 0  [0-10.0%]  (0/35) | 0  [0-13.2%]  (0/26) | 0  [0-14.8%]  (0/23) |
| 9  (Lot-et-Garonne) | Infected area | / | / | 2.0% [0.2-6.9%]  (2/102) | 6.4% [2.1-14.3%]  (5/78) | 12.0% [2.5-31.2%] (3/25) | 11.1% [3.1-26.1%] (4/36) |
|  | Buffer area | / | / | 0  [0-46%] (0/6) | 0  [0-8.6%] (0/41) | 0  [0-16.8%]  (0/20) | 3.1% [0.1-16.2%] (1/32) |
| 10  (Pays Basque) | Infected area | / | / | / | 40%  [5-85%] (2/5) | (0/0) | (0/0) |
|  | Buffer area | / | / | / | (0/0) | 0  [0-60%]  (0/4) | 12.5% [0.3-53%] (1/8) |
| 11  (Ariège/Haute-Garonne) | Infected area | 0 [0-13.2%] (0/26) | 0 [0-6.4%] (0/56) | / | / | 4.2% [0.5-14.3%] (2/48) | 0 [0-8.2%] (0/43) |
|  | Buffer area | / | / | / | / | 0 [0-71%]  (0/3) | 0 [0-20.6%]  (0/16) |

*/: Targeted surveillance in wild boars not required in the area*
