# Supplementary material for: Infection of Wildlife by Mycobacterium bovis in France Assessment Through a National Surveillance System, Sylvatub
Source: Front Vet Sci. 2018 Oct 30;5:262. doi: 10.3389/fvets.2018.00262 (PMC6220493; doi:10.3389/fvets.2018.00262)
Supplement: Supplementary file 3 [file Table_3.doc]

**SUPPLEMENTARY TABLE 3** Apparent prevalence rates in wild boars collected by targeted surveillance by areas from the 2011-2012 to the 2016-2017 hunting season (percentages are given with 95% confidence intervals (CI); in brackets number of infected/analyzed animals)

| **No. of the at-risk area**  **(full name of the area)** | **2011-2012** | **2012-2013** | **2013-2014** | **2014-2015** | **2015-2016** | **2016-2017** |
| --- | --- | --- | --- | --- | --- | --- |
| 1  (Brotonne-Mauny forest) | 1.1% | 0.5% | 2.5% | 2.5% | 2.0% | 0.5% |
|  | [0.1-3.9%] | [0-2.7%] | [0.8-5.8%] | [0.8-5.7%] | [0.6-5.1%] | [0-2.8%] |
|  | (2/185) | (1/204) | (5/197) | (5/201) | (4/197) | (1/197) |
| 2  (Côte-d’Or) | 3.1% | 3.3% | 2.8% | 2.5% | 2.1% | 2.4% |
|  | [1.8-5.1%] | [1.6-6.0%] | [0.9-6.4%] | [0.5-7.3%] | [0.6-5.2%] | [0.8-5.5%] |
|  | (16/508) | (10/303) | (5/180) | (3/118) | (4/194) | (5/210) |
| 3  (Dordogne/Charente/Charente-Maritime/Haute-Vienne/Corrèze/Gironde) | 0.9% | 2.1% | 5.0% | 5.7% | 3.0% | 2.5% |
|  | [0.1-3.3%] | [0.4-6.1%] | [2.8-8.3%] | [3.5-8.7%] | [1.5-5.3%] | [1.2-4.5%] |
|  | (2/214) | (3/140) | (14/279) | (20/348) | (11/370) | (10/400) |
| 4  (Dordogne/Lot) | 0 | 5.0% | 3.7% | 3.0% | 2.4% | 4.1% |
|  | [0-9.5%] | [1.4-12.3%] | [1.0-9.2%] | [0.8-7.5%] | [0.6-5.9%] | [1.7-8.2%] |
|  | (0/37) | (4/80) | (4/108) | (4/134) | (4/169) | (7/172) |
| 5  (Béarn) | 6.2% | 0.9% | 4.3% | 3.6% | 2.4% | 5.9% |
|  | [2.3-13.0%] | [0.1-3.1%] | [1.6-9.2%] | [0.4-12.5%] | [0.5-6.9%] | [2.9-10.6%] |
|  | (6/97) | (2/234) | (6/139) | (2/55) | (3/125) | (10/170) |
| 6  (Ardennes/Marne) | / | / | 0 | 0 | 0 | 0 |
|  |  |  | [0-3.3%] | [0-3.5%] | [0-2.8%] | [0-4.9%] |
|  |  |  | (0/111) | (0/103) | (0/128) | (0/74) |
| 7  (Marne - Reims mountain) | / | / | 0 | 0 | 0 | / |
|  |  |  | [0-1.9%] | [0-2.1%] | [0-3.4%] |  |
|  |  |  | (0/197) | (0/171) | (0/107) |  |
| 8  (Loir-et-Cher) | / | / | / | / | 0 | 0 |
|  |  |  |  |  | [0-0.6%] | [0-1.1%] |
|  |  |  |  |  | (0/662) | (0/324) |
| 9  (Lot-et-Garonne) | / | / | / | 4.0% | 0 | 10.4% |
|  |  |  |  | [0.1-20.4%] | [0-4.3%] | [3.8-20.5%] |
|  |  |  |  | (1/25) | (0/83) | (6/60) |
| 10  (Pays Basque) | / | / | / | / | 0 |  |
|  |  |  |  |  | [0-8.8%] |  |
|  |  |  |  |  | (0/40) | (0/0) |
| 11  (Ariège/Haute-Garonne) | / | / | / | / | 1.1% | 0 |
|  |  |  |  |  | [0-6.0%] | [0-3.7%] |
|  |  |  |  |  | (1/91) | (0/98) |

*/: Targeted surveillance in badgers not required on the area*
