# Supplementary material for: Infection of Wildlife by Mycobacterium bovis in France Assessment Through a National Surveillance System, Sylvatub
Source: Front Vet Sci. 2018 Oct 30;5:262. doi: 10.3389/fvets.2018.00262 (PMC6220493; doi:10.3389/fvets.2018.00262)
Supplement: Supplementary file 4 [file Table_4.doc]

**SUPPLEMENTARY TABLE 4** Apparent prevalence rates in red deer collected by targeted surveillance by areas from the 2011-2012 to the 2016-2017 hunting season (percentages are given with 95% confidence intervals (CI); in brackets number of infected/analyzed animals)

| **No. of the at-risk area**  **(full name of the area)** | **2011-2012** | **2012-2013** | **2013-2014** | **2014-2015** | **2015-2016** | **2016-2017** |
| --- | --- | --- | --- | --- | --- | --- |
| 1*  (Brotonne-Mauny forest) | (0/5) | (0/3) | (0/2) | (0/2) | (0/0) | (0/0) |
| 2*  (Côte-d’Or) | 0.4% | 1.1% | 0 | 0 |  |  |
|  | [0-2.3%] | [0.2-3.2%] | [0-2.4%] | [0-3.8%] |  |  |
|  | (1/243) | (3/270) | (0/154) | (0/95) | (1/3) | (1/4) |
| 3^#^  (Dordogne/Charente/Charente-Maritime/Haute-Vienne/Corrèze/Gironde) | / | / | (7 pme) | (10 pme) | (12 pme) | (33 pme) |
|  |  |  | (0/1) | (0/1) |  |  |
| 4^#^  (Dordogne/Lot) |  |  |  |  |  | 0 |
|  |  |  |  |  |  | [0-12.3%] |
|  |  |  |  | (0/2) | (0/3) | (0/28) |
| 6^#^  (Ardennes/Marne) | / | / | (89 pme) | (81 pme) |  |  |
|  |  |  | (0/1) | (0/2) | (0/0) | (0/0) |
| 7^#^  (Marne - Reims mountain) | / | / | (67 pme) | (32 pme) | / | / |
|  |  |  | (0/2) |  |  |  |
| 8*  (Loir-et-Cher) | / | / |  | / | 0 | 0 |
|  |  |  |  |  | [0-1.0%] | [0-1.3%] |
|  |  |  |  |  | (0/358) | (0/273) |

*pme: number of post-mortem red deer examination;*

*/: Targeted surveillance in red deer not required in the area*

**Areas with systematic analysis*

^#^ *Areas with analysis only in red deer with TB-like lesions*
